# Supplementary material for: Manipulating coordination environment for a high-voltage aqueous copper-chlorine battery
Source: Nat Commun. 2023 Oct 24;14:6738. doi: 10.1038/s41467-023-42549-z (PMC10598032; doi:10.1038/s41467-023-42549-z)
Supplement: Supplementary file 1 — Supplementary Information [file 41467_2023_42549_MOESM1_ESM.pdf]

Supplementary Information

Manipulating coordination environment for a high-voltage aqueous copper-chlorine battery

Xiangyong Zhang[1,2], Hua Wei[1,2], Shizhen Li[1,2], Baohui Ren[1,2], Jingjing Jiang[1,2], Guangmeng Qu[2], Haiming Lv[2], Guojin Liang[3], Guangming Chen[1], Chunyi Zhi[2,3]*, Hongfei Li[4]*, Zhuoxin Liu[1]*

Address

[1] College of Materials Science and Engineering, Shenzhen University, Shenzhen 518055, China

[2] Songshan Lake Materials Laboratory, Dongguan, Guangdong 523808, China

[3] Department of Materials Science and Engineering, City University of Hong Kong, 83 Tat Chee Avenue, Kowloon, Hong Kong 999077, China

[4] School of System Design and Intelligent Manufacturing, Southern University of Science and Technology, Shenzhen 518055, China

E-mail

cy.zhi@cityu.edu.hk; lihf@sustech.edu.cn; liuzhuoxin@szu.edu.cn

Supplementary Figures

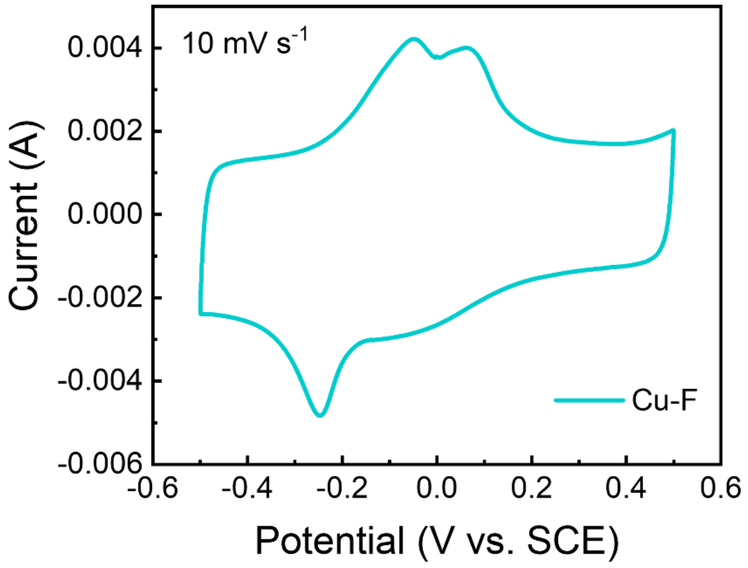

Supplementary Figure 1. The CV curve in Cu-F electrolyte swept at a scan rate of 10 mV s^{-1}. The electrochemical tests were conducted in a three-electrode cell consisting of a carbon felt working electrode, a Pt plate (1 × 1 cm) counter electrode, and an SCE reference electrode.

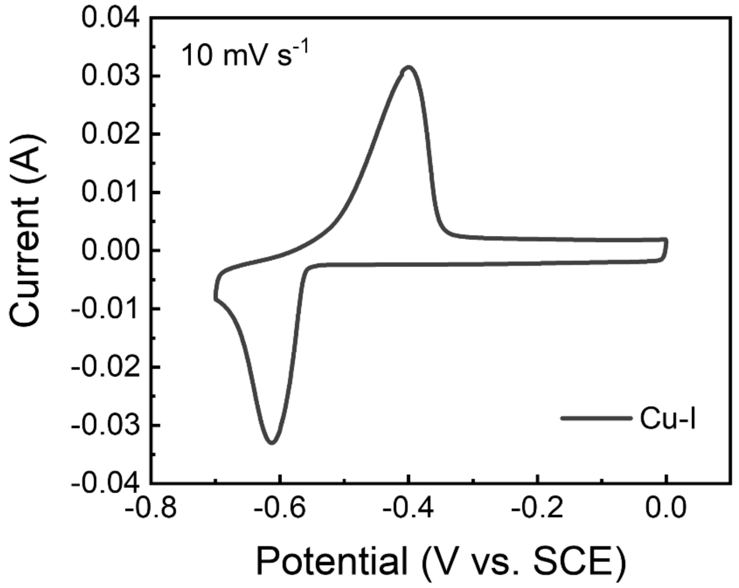

Supplementary Figure 2. The CV curve in Cu-I electrolyte swept at a scan rate of 10 mV s^{-1}. The electrochemical tests were conducted in a three-electrode cell consisting of a carbon felt working electrode, a Pt plate (1 × 1 cm) counter electrode, and an SCE reference electrode.

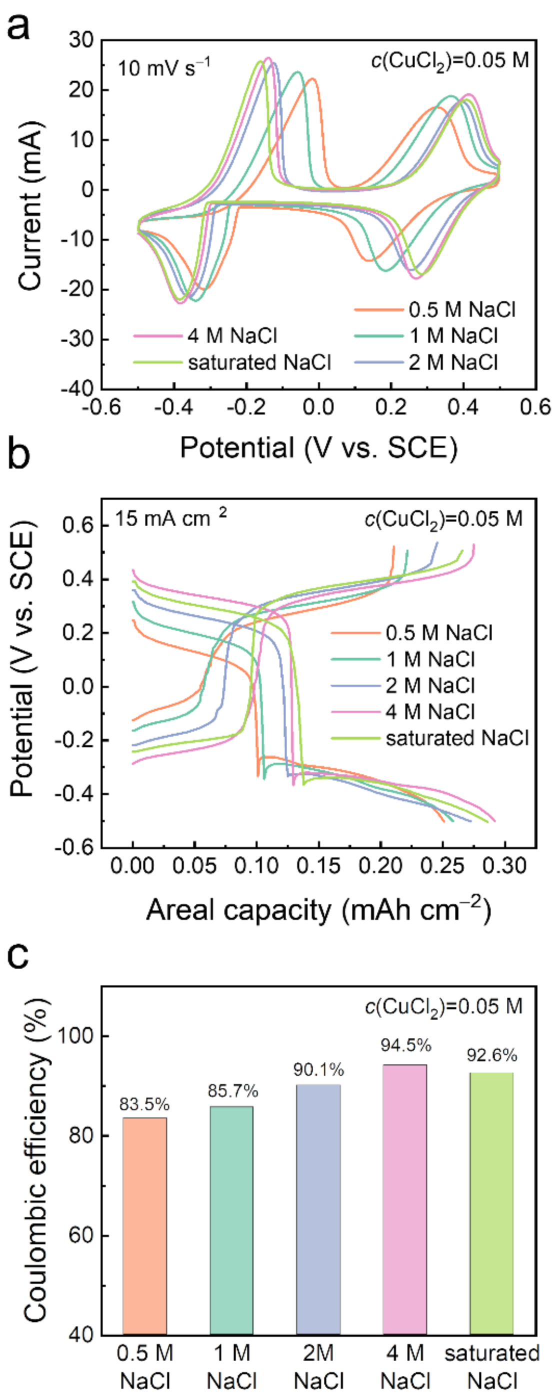

Supplementary Figure 3. The influences of NaCl concentration. (a) CV curves, (b) GCD curves, and (c) the Coulombic efficiency with varying NaCl concentrations. The electrochemical tests were conducted in a three-electrode cell consisting of a carbon felt working electrode, a Pt plate (1 × 1 cm) counter electrode, and an SCE reference electrode.

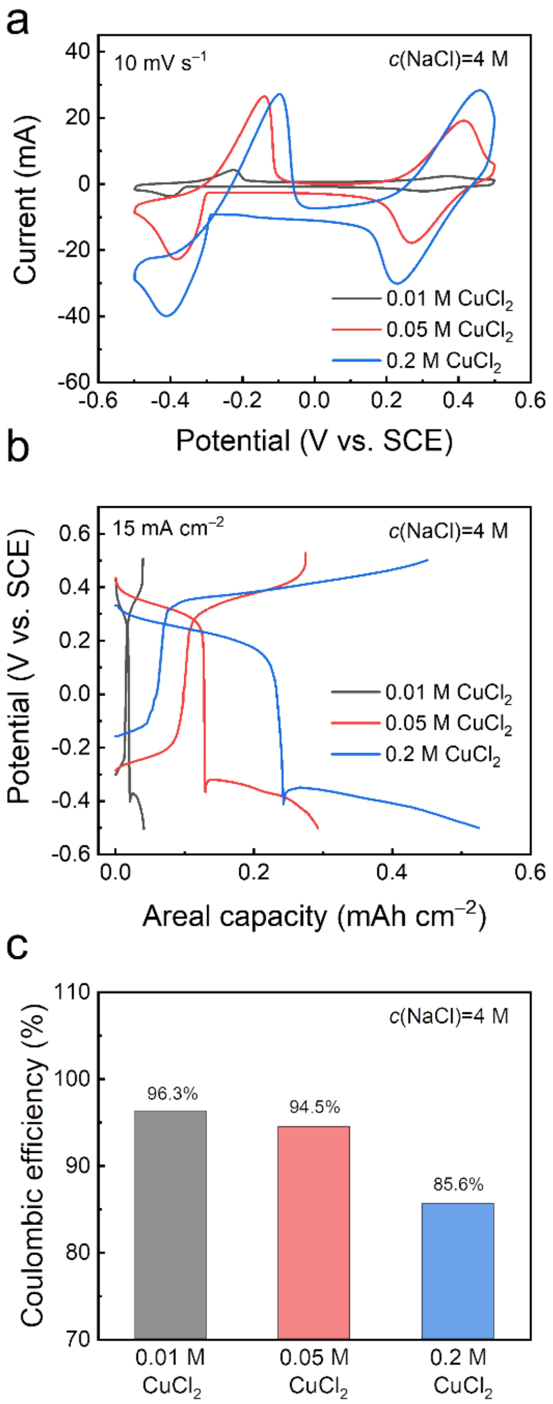

Supplementary Figure 4. The influences of $CuCl_2$ concentration. (a) CV curves, (b) GCD curves, and (c) the Coulombic efficiency with varying $CuCl_2$ concentrations. The electrochemical tests were conducted in a three-electrode cell consisting of a carbon felt working electrode, a Pt plate (1 × 1 cm) counter electrode, and an SCE reference electrode.

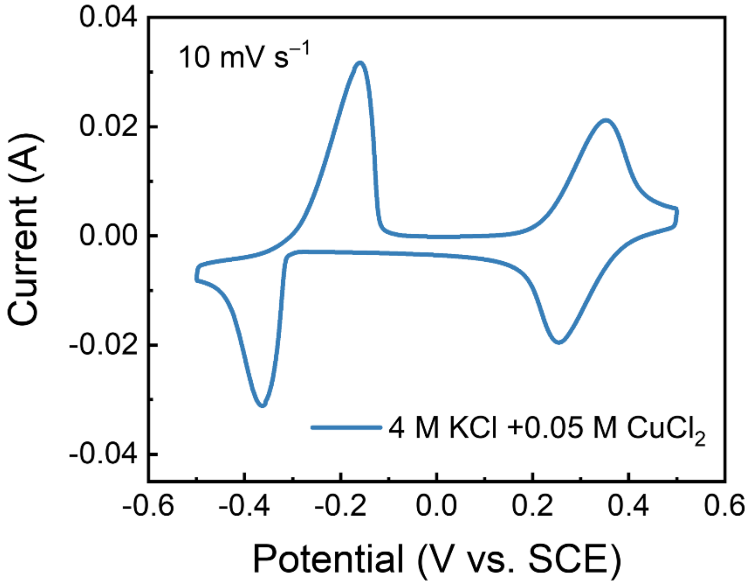

Supplementary Figure 5. The CV curve in 4 M KCl + 0.05 M $CuCl_2$ swept at a scan rate of 10 mV s^{-1}. The electrochemical tests were conducted in a three-electrode cell consisting of a carbon felt working electrode, a Pt plate (1 × 1 cm) counter electrode, and an SCE reference electrode.

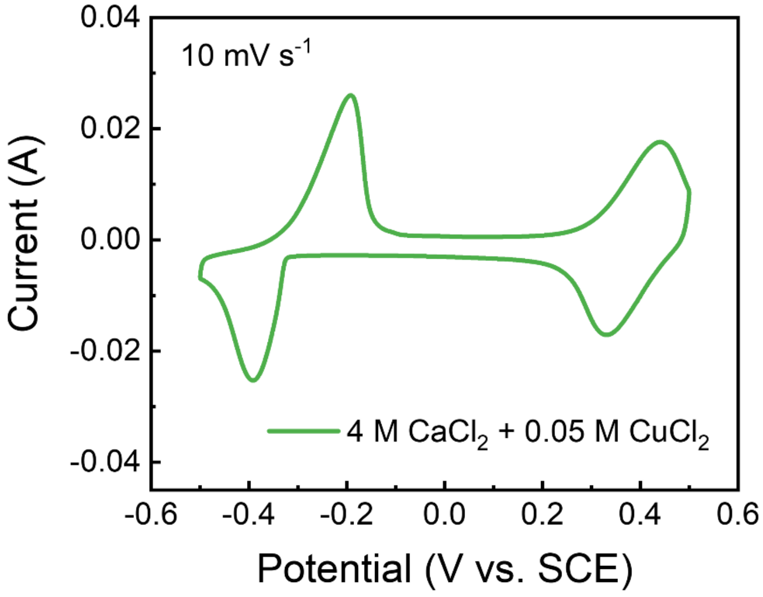

Supplementary Figure 6. The CV curve in 4 M $CaCl_2$ + 0.05 M $CuCl_2$ swept at a scan rate of 10 mV s^{-1}. The electrochemical tests were conducted in a three-electrode cell consisting of a carbon felt working electrode, a Pt plate (1 × 1 cm) counter electrode, and an SCE reference electrode.

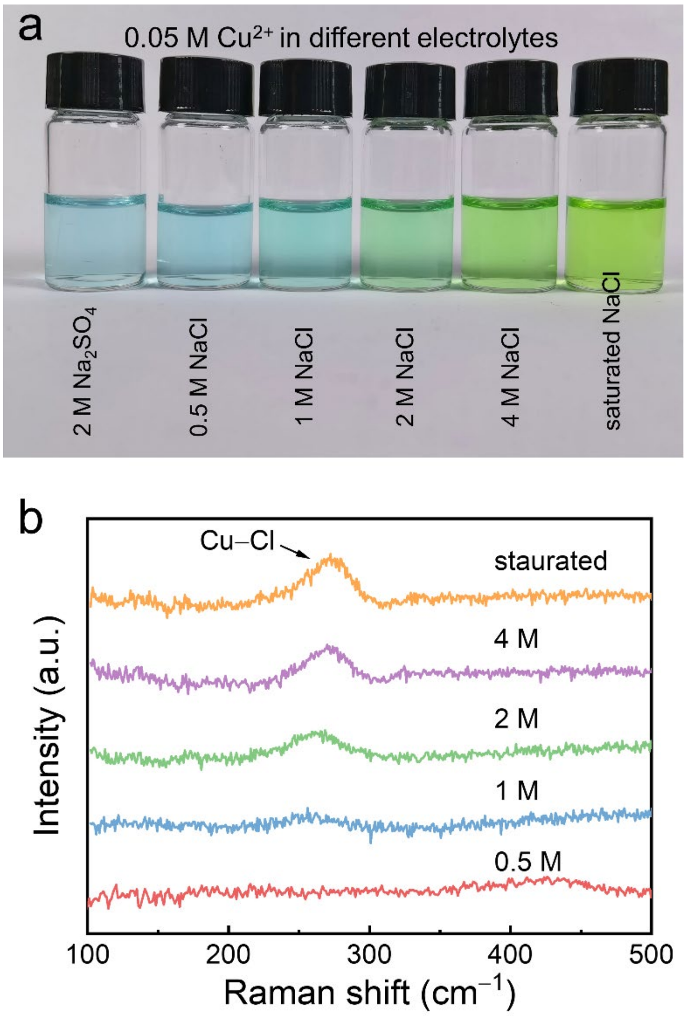

Supplementary Figure 7. The Cu−Cl complex coordination. (a) Optical photos of $CuCl_2$ in different electrolytes. (b) The SERS spectra of $CuCl_2$ in various NaCl solutions.

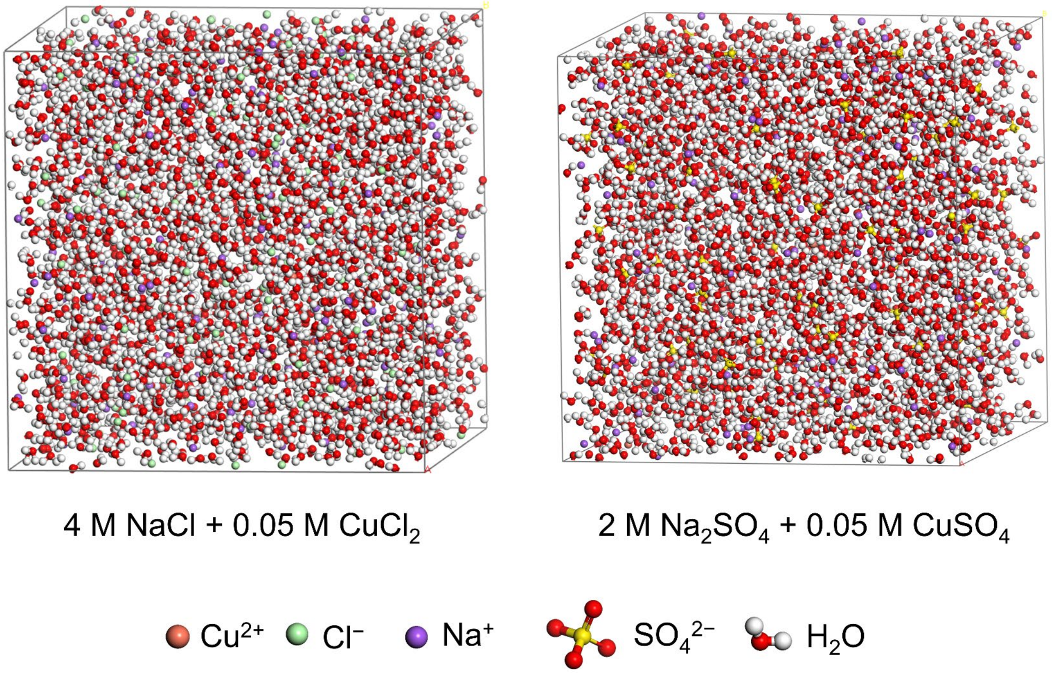

Supplementary Figure 8. Snapshots of the simulation box for Cu−Cl and Cu−H_2O electrolytes.

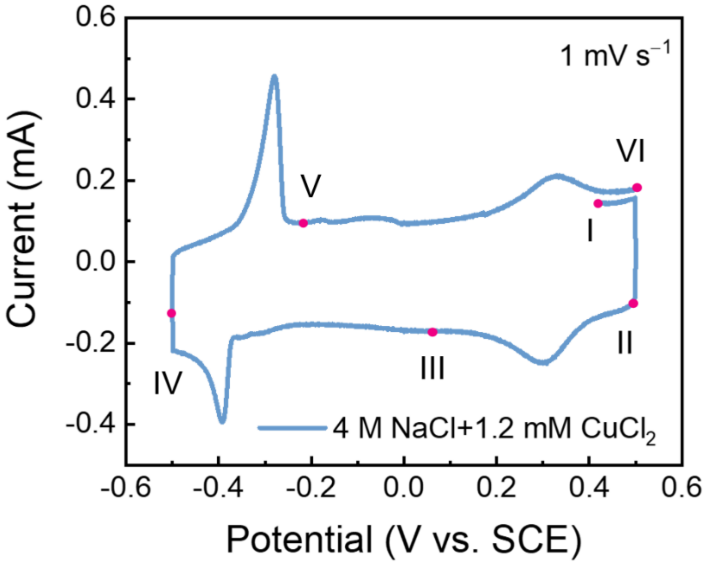

Supplementary Figure 9. The CV curve in 4 M NaCl + 1.2 mM $CuCl_2$ swept at a scan rate of 1 mV s^{-1}. The electrochemical tests were conducted in a three-electrode cell consisting of a carbon felt working electrode, a Pt plate (1 × 1 cm) counter electrode, and an SCE reference electrode.

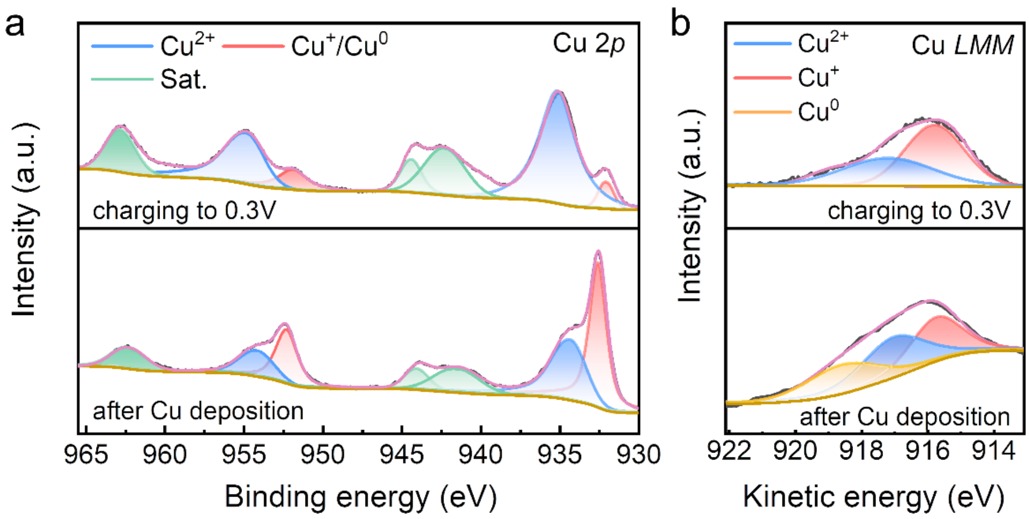

Supplementary Figure 10. Electrode surface valence changes during the electrochemical processes. (a) XPS spectra and (b) XAES spectra corresponding to charging to 0.3 V and after Cu deposition.

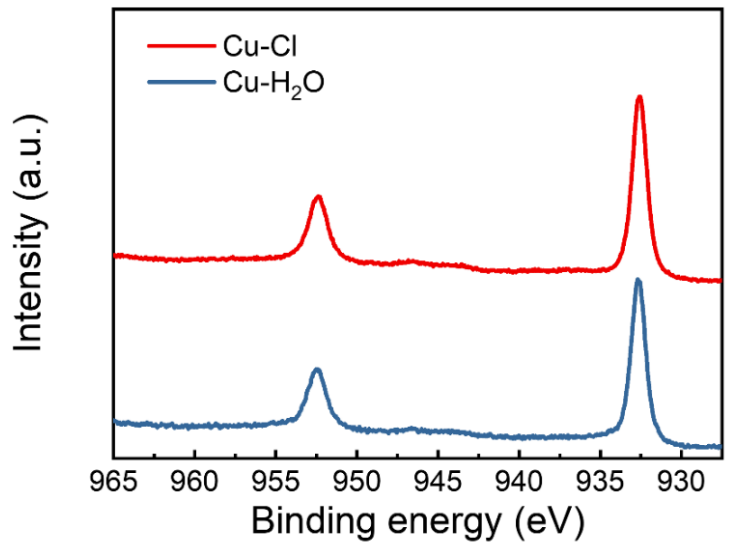

Supplementary Figure 11. XPS spectra of copper deposited in Cu−Cl and Cu−H_2O electrolytes.

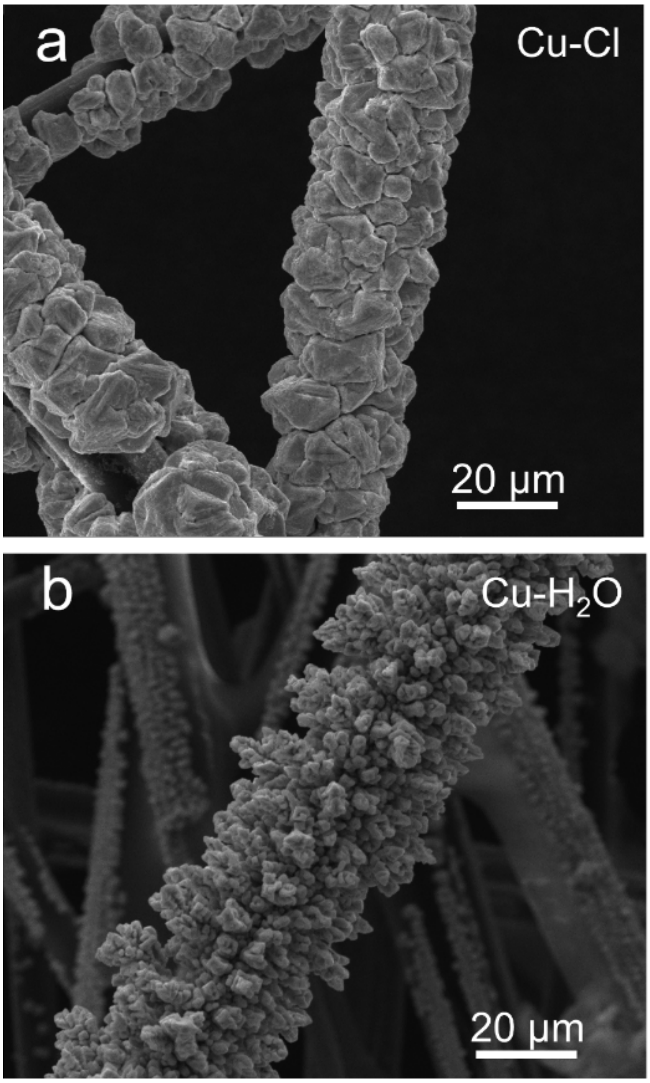

Supplementary Figure 12. SEM images of copper deposited. (a) Cu−Cl electrolyte, and (b) Cu−H_2O electrolyte. Copper deposition was achieved through discharging at a constant current of 10 mA cm^{-2}. The amounts of copper deposited in Cu−Cl and Cu−H_2O electrolytes were calculated to be 0.37 mg cm^{-2} and 0.24 mg cm^{-2}, respectively. The discharging tests were conducted in a three-electrode cell consisting of a carbon felt working electrode, a Pt plate (1 × 1 cm) counter electrode, and an SCE reference electrode.

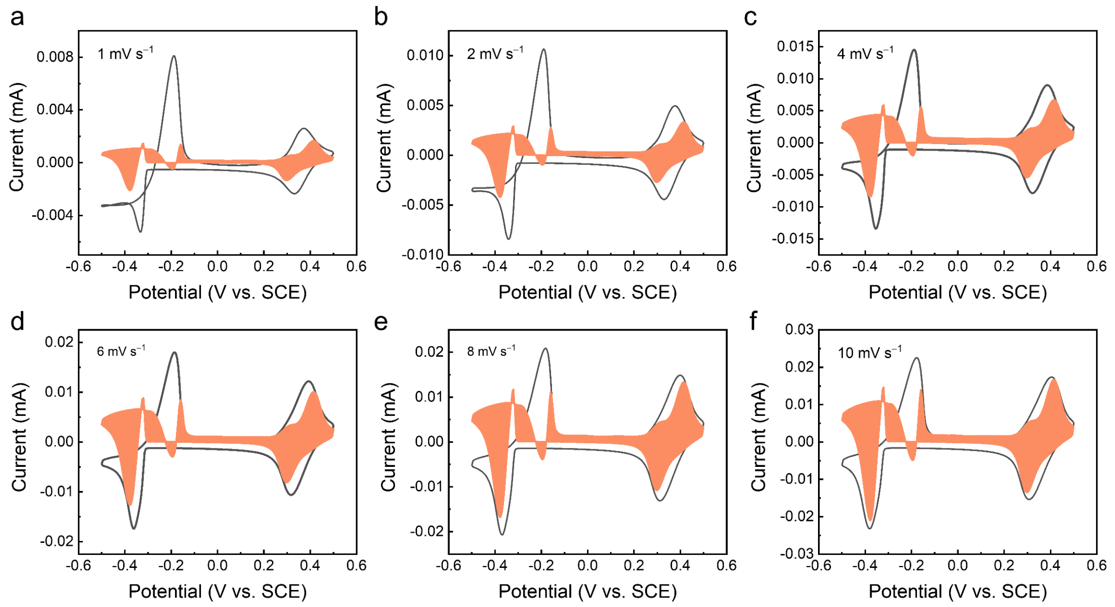

Supplementary Figure 13. The CV curves for Cu−Cl electrolyte at various scan rates (the shaded areas show the calculated capacitive contributions). (a) 1 mV s^{-1}. (b) 2 mV s^{-1}. (c) 4 mV s^{-1}. (d) 6 mV s^{-1}. (e) 8 mV s^{-1}. (f) 10 mV s^{-1}. The electrochemical tests were conducted in a three-electrode cell consisting of a carbon felt working electrode, a Pt plate (1 × 1 cm) counter electrode, and an SCE reference electrode.

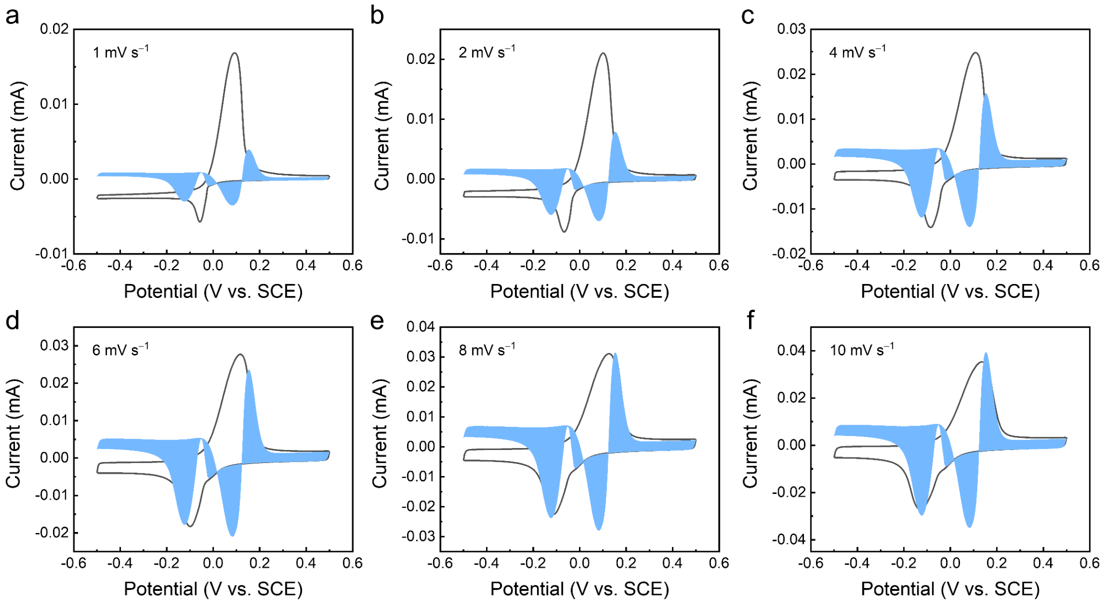

Supplementary Figure 14. The CV curves for Cu−H_2O electrolyte at various scan rates (the shaded areas show the calculated capacitive contributions). (a) 1 mV s^{-1}. (b) 2 mV s^{-1}. (c) 4 mV s^{-1}. (d) 6 mV s^{-1}. (e) 8 mV s^{-1}. (f) 10 mV s^{-1}. The electrochemical tests were conducted in a three-electrode cell consisting of a carbon felt working electrode, a Pt plate (1 × 1 cm) counter electrode, and an SCE reference electrode.

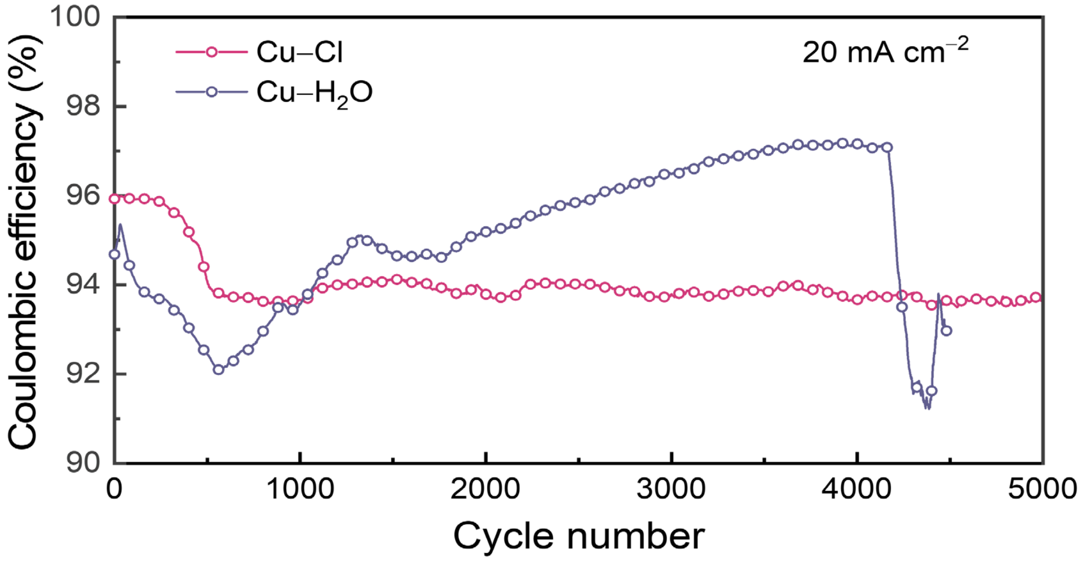

Supplementary Figure 15. CE curves in Cu−Cl and Cu−H_2O electrolytes. These CE curves correspond to the long-term stability test in Cu−Cl and Cu−H_2O electrolytes at 20 mA cm^{-2} shown in Figure 4h.

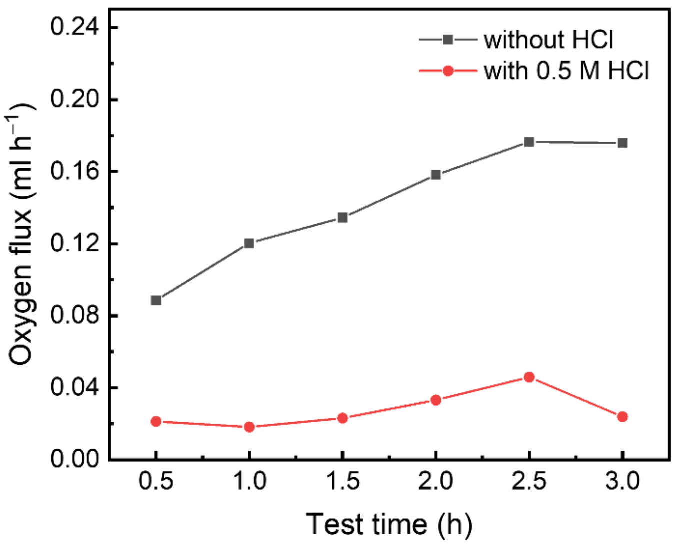

Supplementary Figure 16. In situ monitoring of oxygen production on an in situ gas chromatography system. The in situ testing system comprises an electrochemical workstation, a three-electrode cell, and a gas chromatograph. A curve of current vs. time was continuously recorded over a 3-hour period using a voltage of 1.3 V (*vs.* SCE) to measure oxygen levels. Data collection occurred every half hour, and the gas flow rate was maintained at 9 ml h^{-1} throughout the testing.

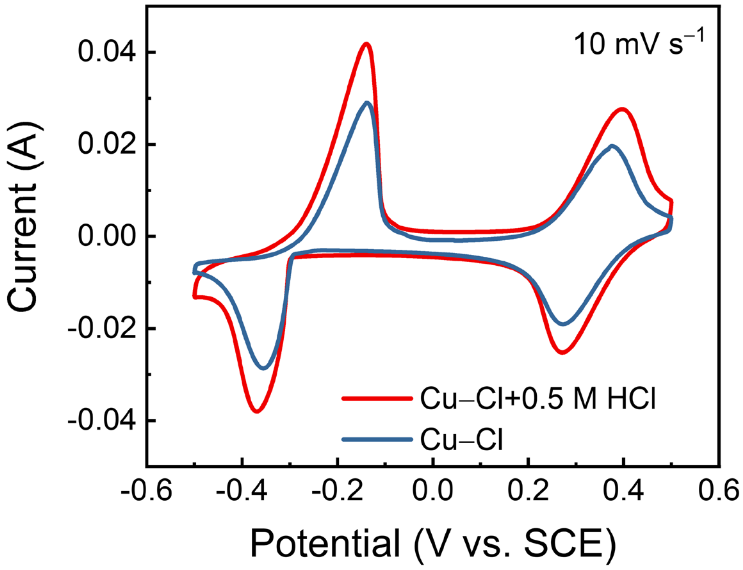

Supplementary Figure 17. The CV curves in Cu−Cl and Cu−Cl + 0.5 M HCl swept at a scan rate of 10 mV s^{-1}. The electrochemical tests were conducted in a three-electrode cell consisting of a carbon felt working electrode, a Pt plate (1 × 1 cm) counter electrode, and an SCE reference electrode.

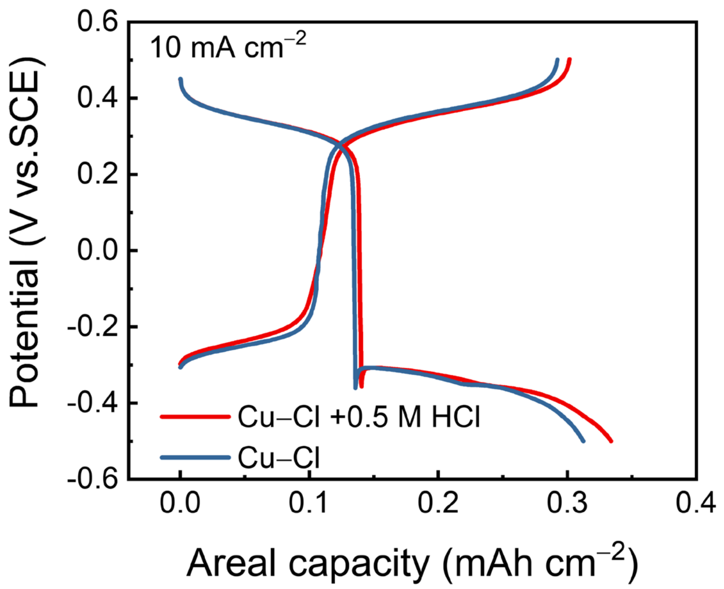

Supplementary Figure 18. The GCD curves in Cu−Cl and Cu−Cl + 0.5 M HCl at a current density of 10 mA cm^{-2}. The electrochemical tests were conducted in a three-electrode cell consisting of a carbon felt working electrode, a Pt plate (1 × 1 cm) counter electrode, and an SCE reference electrode.

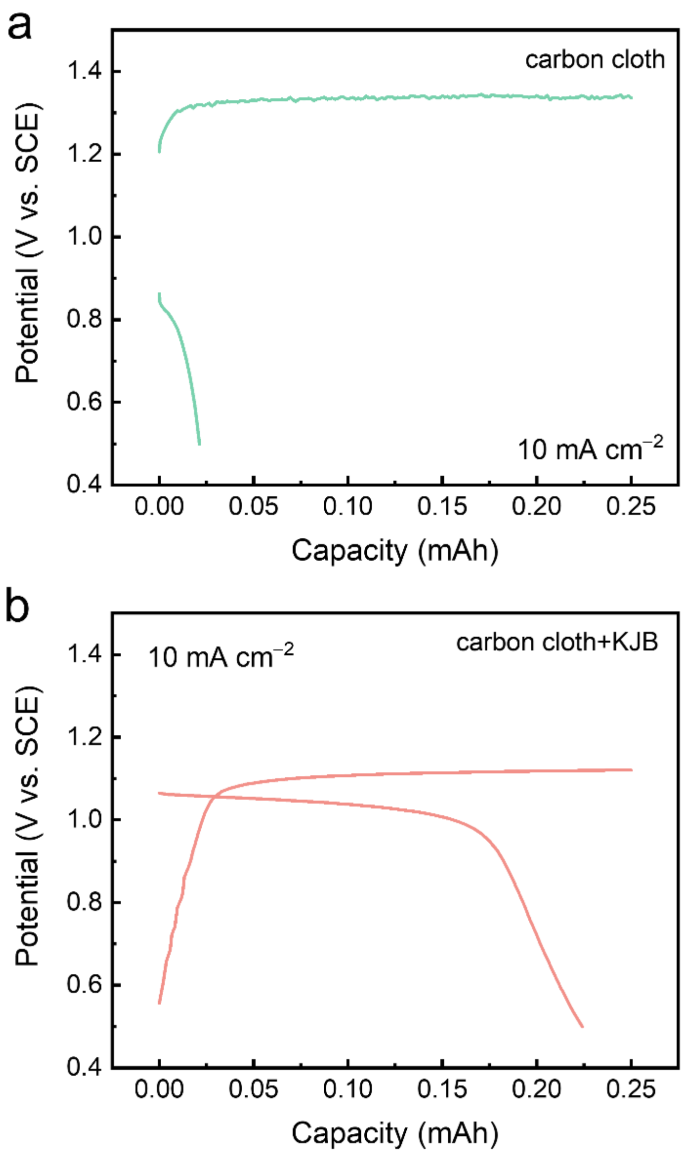

Supplementary Figure 19. Contribution of carbon cloth to the capacity. (a) GCD curve of pure carbon cloth at 10 mA cm^{-2}. (b) GCD curve of carbon cloth + KJB at 10 mA cm^{-2}. The electrochemical tests were conducted in a three-electrode cell consisting of a carbon felt working electrode, a Pt plate (1 × 1 cm) counter electrode, and an SCE reference electrode.

Supplementary Tables

Supplementary Table 1 The comparison of discharge voltage between this work and other reported aqueous Cu-based batteries.

Full cell	Discharge voltage (V)
Cu-PBA[1]	0.75
Cu-S[2]	0.15
Cu-MnO_2[3]	0.95
Cu-MnO_2[4]	0.98
Zn-Cu[5]	0.75
Zn-Cu[6]	0.8
Zn-Cu[7]	0.7
Zn-Cu[8]	0.93
**This work**	**1.3**

Supplementary Note 1. To ensure the reproducibility of results, each CV test was carried out on a minimum of 5 cells, each GCD test was performed on over 20 cells, and at least 10 cells were utilized for each cycling measurement.

Supplementary References

1. Liang G*, et al.* Commencing an acidic battery based on a copper anode with ultrafast proton-regulated kinetics and superior dendrite-free property. *Adv. Mater.* **31**, 1905873 (2019).
2. Yan H*, et al.* Controllable CN site assisting observable potential difference for homogeneous copper deposition in aqueous Cu-S batteries. *Energy Stor. Mater.* **48**, 74-81 (2022).
3. Wang M*, et al.* Electrode-less MnO_2-metal batteries with deposition and stripping chemistry. *Small* **17**, 2103921 (2021).
4. Liang G*, et al.* A universal principle to design reversible aqueous batteries based on deposition–dissolution mechanism. *Adv. Energy Mater.* **9**, 1901838 (2019).
5. Zhu Q*, et al.* Realizing a Rechargeable High-Performance Cu–Zn Battery by Adjusting the Solubility of Cu^{2+}. *Adv. Funct. Mater.* **29**, 1905979 (2019).
6. Mypati S, Khazaeli A, Barz DP. A novel rechargeable zinc–copper battery without a separator. *J. Energy Stor.* **42**, 103109 (2021).
7. Jameson A, Khazaeli A, Barz DP. A rechargeable zinc copper battery using a selective cation exchange membrane. *J. Power Sources* **453**, 227873 (2020).
8. Zhang H*, et al.* Using Li^+ as the electrochemical messenger to fabricate an aqueous rechargeable Zn–Cu battery. *Chem. Commun.* **51**, 7294-7297 (2015).
