# Supplementary material for: Manipulating coordination environment for a high-voltage aqueous copper-chlorine battery
Source: Nat Commun. 2023 Oct 24;14:6738. doi: 10.1038/s41467-023-42549-z (PMC10598032; doi:10.1038/s41467-023-42549-z)
Supplement: Supplementary file 3 — Reporting Summary [file 41467_2023_42549_MOESM3_ESM.pdf]

nature portfolio

Corresponding author(s): Zhuoxin Liu

Last updated by author(s): Oct 7, 2023

Reporting Summary

Nature Portfolio wishes to improve the reproducibility of the work that we publish. This form provides structure for consistency and transparency in reporting. For further information on Nature Portfolio policies, see our Editorial Policies and the Editorial Policy Checklist.

Statistics

For all statistical analyses, confirm that the following items are present in the figure legend, table legend, main text, or Methods section.

n/a	Confirmed	
☒	☐	The exact sample size (n) for each experimental group/condition, given as a discrete number and unit of measurement
☐	☒	A statement on whether measurements were taken from distinct samples or whether the same sample was measured repeatedly
☒	☐	The statistical test(s) used AND whether they are one- or two-sided<br>*Only common tests should be described solely by name; describe more complex techniques in the Methods section.*
☒	☐	A description of all covariates tested
☒	☐	A description of any assumptions or corrections, such as tests of normality and adjustment for multiple comparisons
☒	☐	A full description of the statistical parameters including central tendency (e.g. means) or other basic estimates (e.g. regression coefficient) AND variation (e.g. standard deviation) or associated estimates of uncertainty (e.g. confidence intervals)
☒	☐	For null hypothesis testing, the test statistic (e.g. F, t, r) with confidence intervals, effect sizes, degrees of freedom and P value noted<br>*Give P values as exact values whenever suitable.*
☒	☐	For Bayesian analysis, information on the choice of priors and Markov chain Monte Carlo settings
☒	☐	For hierarchical and complex designs, identification of the appropriate level for tests and full reporting of outcomes
☒	☐	Estimates of effect sizes (e.g. Cohen's d, Pearson's r), indicating how they were calculated

Our web collection on statistics for biologists contains articles on many of the points above.

Software and code

Policy information about availability of computer code

Data collection	The XRD patterns were recorded on a Mini Flex 600 X-ray diffractometer (Rigaku). The scanning electron microscope (SEM) images were taken on a VEGA3 SEM. The XPS and XAES was measured on the Thermo Fisher ESCALAB 250Xi system. The UV–VIS spectra were collected on a Lambda 365 UV–VIS spectrophotometer. The SERS spectra were recorded using the Raman spectrometer (Thermo Fischer DXR). NMR spectra were collected with Bruker AVANCE Ⅲ HD 400MHz. The cyclic voltammetry (CV) tests were operated with a CHI 700E electrochemical workstation. The galvanostatic charge-discharge rate capability and battery cycling performance were carried out on a Neware battery test system. Calculation data was based on Materials studio and Guassian 09 program.
Data analysis	powerpoint, origin

For manuscripts utilizing custom algorithms or software that are central to the research but not yet described in published literature, software must be made available to editors and reviewers. We strongly encourage code deposition in a community repository (e.g. GitHub). See the Nature Portfolio guidelines for submitting code & software for further information.

Data

Policy information about availability of data

All manuscripts must include a data availability statement. This statement should provide the following information, where applicable:

- Accession codes, unique identifiers, or web links for publicly available datasets
- A description of any restrictions on data availability
- For clinical datasets or third party data, please ensure that the statement adheres to our policy

The data that support the findings of this study are available within the text including the Methods, and Supplemental information. Raw datasets related to the current work are available from the corresponding author on reasonable request.

Research involving human participants, their data, or biological material

Policy information about studies with human participants or human data. See also policy information about sex, gender (identity/presentation), and sexual orientation and race, ethnicity and racism.

Reporting on sex and gender	The findings apply to all sex and gender. No sex and gender were considered in study design.
Reporting on race, ethnicity, or other socially relevant groupings	There are no data about the race, ethnicity, or other socially relevant groupings in our research.
Population characteristics	Not applicable.
Recruitment	Not applicable.
Ethics oversight	Not applicable.

Note that full information on the approval of the study protocol must also be provided in the manuscript.

Field-specific reporting

Please select the one below that is the best fit for your research. If you are not sure, read the appropriate sections before making your selection.

☐ Life sciences ☐ Behavioural & social sciences ☒ Ecological, evolutionary & environmental sciences

For a reference copy of the document with all sections, see nature.com/documents/nr-reporting-summary-flat.pdf

Ecological, evolutionary & environmental sciences study design

All studies must disclose on these points even when the disclosure is negative.

Study description	Manipulating coordination environment of Cu ions enables a high-voltage aqueous Cu-Cl2 battery
Research sample	Copper chloride(CuCl2), sodium chloride(NaCl), Ketjen Black, carbon cloth, hydrochloric acid(HCl), sodium sulfate (Na2SO4), copper sulfate (CuSO4·5H2O)
Sampling strategy	The chlorine coordination was achieved using an aqueous solution comprising 4 M NaCl and 0.05 M CuCl2 as the electrolyte (denoted Cu–Cl electrolyte).The water coordination was achieved using a solution comprising 2 M Na2SO4 and 0.05 M CuSO4 as the electrolyte (denoted Cu–H2O electrolyte).<br>Preparation of the Ketjen Black (KJB) electrode. Ninety percent by weight of Ketjen Black, five percent by weight of sodium carboxymethyl cellulose (CMC) and five percent by weight of styrene-butadiene rubber (SBR) were mixed and grinded with deionized water to make a homogeneous slurry. Then the mixed paste was cast on carbon cloth current collectors.
Data collection	The XRD patterns were recorded on a Mini Flex 600 X-ray diffractometer (Rigaku). The scanning electron microscope (SEM) images were taken on a VEGA3 SEM. The XPS and XAES was measured on the Thermo Fisher ESCALAB 250Xi system. The UV–VIS spectra were collected on a Lambda 365 UV–VIS spectrophotometer. The SERS spectra were recorded using the Raman spectrometer (Thermo Fischer DXR). NMR spectra were collected with Bruker AVANCE Ⅲ HD 400MHz. The cyclic voltammetry (CV) tests were operated with a CHI 700E electrochemical workstation. The galvanostatic charge-discharge rate capability and battery cycling performance were carried out on a Neware battery test system. Calculation data was based on Materials studio and Guassian 09 program.
Timing and spatial scale	Oct. 2021--Sep.2022
Data exclusions	No data were excluded.
Reproducibility	All attempts to repeat the experiment were successful.

Randomization	Not applicable.
Blinding	Not applicable.

Did the study involve field work? ☐ Yes ☒ No

Reporting for specific materials, systems and methods

We require information from authors about some types of materials, experimental systems and methods used in many studies. Here, indicate whether each material, system or method listed is relevant to your study. If you are not sure if a list item applies to your research, read the appropriate section before selecting a response.

Materials & experimental systems

n/a	Involved in the study	
☒	☐	Antibodies
☒	☐	Eukaryotic cell lines
☒	☐	Palaeontology and archaeology
☒	☐	Animals and other organisms
☒	☐	Clinical data
☒	☐	Dual use research of concern
☒	☐	Plants

Methods

n/a	Involved in the study	
☒	☐	ChIP-seq
☒	☐	Flow cytometry
☒	☐	MRI-based neuroimaging
